# Total Hepatitis B Core Antigen Antibody, a Quantitative Non-Invasive Marker of Hepatitis B Virus Induced Liver Disease

**DOI:** 10.1371/journal.pone.0130209

**Published:** 2015-06-26

**Authors:** Quan Yuan, Liu-Wei Song, Daniela Cavallone, Francesco Moriconi, Beatrice Cherubini, Piero Colombatto, Filippo Oliveri, Barbara Agata Coco, Gabriele Ricco, Ferruccio Bonino, James Wai Kuo Shih, Ning-Shao Xia, Maurizia Rossana Brunetto

**Affiliations:** 1 State Key Laboratory of Molecular Vaccinology and Molecular Diagnostics, National Institute of Diagnostics and Vaccine Development in Infectious Diseases, School of Public Health, Xiamen University, Xiamen, China; 2 Laboratory of Molecular Genetics and Pathology of Hepatitis Viruses, Hepatology Unit, Reference Center of the Tuscany Region for Chronic Liver Disease and Cancer, University Hospital of Pisa, Pisa, Italy; 3 Digestive and Liver Disease, General Medicine II Unit, University Hospital of Pisa, Pisa, Italy; Harvard Medical School, UNITED STATES

## Abstract

Non invasive immunologic markers of virus-induced liver disease are unmet needs. We tested the clinical significance of quantitative total and IgM-anti-HBc in well characterized chronic-HBsAg-carriers. Sera (212) were obtained from 111 HBsAg-carriers followed-up for 52 months (28-216) during different phases of chronic-HBV-genotype-D-infection: 10 HBeAg-positive, 25 inactive-carriers (HBV-DNA≤2000IU/ml, ALT<30U/L), 66 HBeAg-negative-CHB-patients and 10 with HDV-super-infection. In 35 patients treated with Peg-IFN±nucleos(t)ide-analogues (NUCs) sera were obtained at baseline, end-of-therapy and week-24-off-therapy and in 22 treated with NUCs (for 60 months, 42-134m) at baseline and end-of-follow-up. HBsAg and IgM-anti-HBc were measured by Architect-assays (Abbott, USA); total-anti-HBc by double-antigen-sandwich-immune-assay (Wantai, China); HBV-DNA by COBAS-TaqMan (Roche, Germany). Total-anti-HBc were detectable in all sera with lower levels in HBsAg-carriers without CHB (immune-tolerant, inactive and HDV-superinfected, median 3.26, range 2.26-4.49 Log_10_ IU/ml) versus untreated-CHB (median 4.68, range 2.76-5.54 Log_10_ IU/ml), p<0.0001. IgM-anti-HBc positive using the chronic-hepatitis-cut-off" (0.130-S/CO) were positive in 102 of 212 sera (48.1%). Overall total-anti-HBc and IgM-anti-HBc correlated significantly (p<0.001, r=0.417). Total-anti-HBc declined significantly in CHB patients with response to Peg-IFN (p<0.001) and in NUC-treated patients (p<0.001); the lowest levels (median 2.68, range 2.12-3.08 Log_10_ IU/ml) were found in long-term responders who cleared HBsAg subsequently. During spontaneous and therapy-induced fluctuations of CHB (remissions and reactivations) total- and IgM-anti-HBc correlated with ALT (p<0.001, r=0.351 and p=0.008, r=0.185 respectively). Total-anti-HBc qualifies as a useful marker of HBV-induced-liver-disease that might help to discriminate major phases of chronic HBV infection and to predict sustained response to antivirals.

## Introduction

Hepatitis-B-Virus (HBV) infection elicits a prominent immune response against hepatitis-B-core-antigen (HBcAg) [1–5]: IgG-antibodies against HBcAg (anti-HBc) persist lifelong in HBV exposed individuals whereas IgM-antibodies decline becoming undetectable after recovery from hepatitis-B [4,6–8]. Commercial assays for IgM-anti-HBc are proposed for diagnosis of acute-hepatitis-B (AHB) that is based on a clinically standardized threshold of 600 Paul-Ehrlich (PEI) [6–9]. Using the analytical sensitivity cut-off of the assays low levels of IgM-anti-HBc were found to fluctuate in chronic-hepatitis-B (CHB) paralleling the disease remission and reactivation phases [10–15]. This prompted the use of different cut-offs values for acute and chronic hepatitis B [10–13]. However, the quantification range of commercially available IgM-anti-HBc assays, specifically designed for diagnosis of AHB hampers the diagnostic accuracy of detecting low antibody levels in chronic HBsAg-carriers [14–17]. Thus, a satisfactory non-invasive marker of HBV-induced liver disease in chronic HBV infection remains an unmet need. In clinical practice the combined quantification of serum HBV-DNA and HBsAg is currently used to discriminate HBeAg-negative-CHB from inactive infection and to monitor the switch from CHB to inactive-carrier during antiviral therapy [17–20]. However, in HBeAg-negative CHB patients undergoing antiviral treatment HBsAg kinetics vary according to HBV genotype and genotype-specific monitoring timeframes and end-of-treatment thresholds have yet to be standardized for a proper response-guided treatment 19]. Recently a new assay based on double antigen sandwich method that detects total (IgG and IgM) anti-HBc was developed and proposed to monitor patients, chronically infected with genotype B and C HBV undergoing antiviral therapy [21–24]. We tested this assay in a validation study on a panel of sera from well characterized genotype D, HBsAg carriers.

## Materials and Methods

Sera (212) were obtained from 111 chronic-HBsAg-carriers infected with HBV-genotype-D (Table 1) followed-up (mean 52-months, 28-216-months). Patients gave an informed consent and the study was approved by the Ethic Committee of the University Hospital of Pisa. All of them were followed with blood tests every 3–6 months, but those with low HBV-DNA levels (<20000 IU/mL) and normal transaminases (ALT) at presentation, underwent monthly testing for at least 1-year for characterization. HBsAg-carriers were classified according to their biochemical and viral profiles [9,17].

**Table 1:** Characteristics of HBsAg Carriers.

HBsAgCarriers	N.ofcases	N.ofsera	Age median(range)	Male/Female	ALTU/Lmedian(range)	HBsAgLog_10_ IU/mlmedian(range)	HBV-DNALog_10_ IU/mlmedian(range)
A: HBeAg-positive	10	10	38.92(21.45–71.04)	8/2	77.5(26–346)	4.99(3.71–5.46)	7.83(0–8.26)
B: HBeAg-negative, inactive	25	25	53.78(31.15–79.41)	17/8	18.0(10–60)	1.72(-1.7–3.22)	1.82(0–3.21)
C: HBeAg-negative, with HDV superinfection	10	10	34.03(13.65–59.86)	9/1	104.5(70–207)	4.17(4.07–4.53)	1.58(0–1.98)
D: HBeAg-negative, chronic hepatitis B	66	167	54.18(32.62–83.52)	45/21	73.0(7–1243)	3.51(-0.38–4.7)	5.08(0–8.13)

A) 10 HBeAg-positive HBsAg-carriers: 9 HBeAg-positive-CHB patients and 1 in the "immune tolerance" phase;

B) 25 inactive-carriers [IC, HBV-DNA levels persistently ≤ 2000 IU/ml and normal ALT (<30 U/L)];

C) 10 HBeAg-negative/anti-HBe positive/anti-HDV-positive carriers with chronic-Delta-hepatitis (CDH);

D) 66 active carriers, HBeAg-negative/anti-HBe-positive CHB with fluctuating HBV-DNA above 20000 IU/mL and elevated ALT.

Sera were obtained once from HBeAg-positive carriers, inactive carriers (IC) and chronic hepatitis Delta (CDH) and at different time points in HBeAg-negative-CHB. Five HBeAg-negative-CHB patients with fluctuating disease profile [20] were tested during transient spontaneous remissions (ALT range 19-28-U/L; HBV-DNA 5.1 x 10^3^–3.7 x 10^4^-IU/ml) and reactivations (ALT range 210-550-U/L; HBV-DNA 2.2 10^4^ x -1.5 x 10^7^-IU/ml). Fifty-seven HBeAg-negative-CHB underwent antiviral treatment: 35 with Peg-IFN-180μg/w±nucleos(t)ide-analogues (NUC) for 12-months and 22 with NUC for a mean of 60-months (range 42-134-months). In Peg-IFN treated patients, HBV-DNA<2000-IU/ml identified virologic response at end-of-treatment (EOT); its persistence at every 3-months for at least 12-months after EOT identified sustained-virologic-response, SVR. Patients with HBV-DNA<2000-IU/ml at EOT, but with recurrence of florid viral-replication thereafter were defined Relapsers (REL); Non-responders (NR) had HBV-DNA levels >2000-IU/ml at EOT. Sera were obtained at baseline (BL) before therapy, EOT and week-24 after EOT (Post-T-FU) in 35 Peg-IFN-treated patients. In 22 NUC (Entecavir or Tenofovir)-treated patients sera were obtained at BL and last-available follow-up sampling, at least 6-months after NUCs discontinuation (Post-T-FU) in patients who stopped therapy.

### Serological tests

HBsAg, anti-HBs, anti-HBc, HBeAg and anti-HBe, anti-HCV, anti-HDV and anti-HIV were detected by commercially immunoassays (Abbott-Laboratories, N-Chicago, USA). IgM-anti-HBc and HBsAg were quantified by Architect-anti-HBc-CMIA and HBsAg-assay (Abbott Laboratories). For quantification of IgM-anti-HBc we used both the AHB cut-off (1 S/CO) and a CHB threshold defined as follows. Architect CMIA and Axsym CORE-M, MEIA immunoassay [using the CHB cut-off: 0.200 IMx-index, proven to discriminate IC and CHB (10)] were compared testing consecutive sera (1050) from HBsAg-carriers with and without liver disease and controls (without HBV-markers) using the Paul-Ehrlich-Institute (Germany) calibration standard curve (0-100-PEI Units). By receiver-operating-characteristic (ROC) curves, the Architect cut-off for CHB was 0.130-S/CO (AUC = 0,716, SE = 0.0189). The analytical concordance between Axsym and Architect assays was highly significant (p<0.0001, r = 0.7737, 95%Cl0.7482–0.7969). HBsAg was quantified as previously reported [17]. Serum HBV DNA levels were quantified by COBAS-TaqMan-assay, sensitivity 6-IU/ml, dynamic-range 6–1.10x 10^8^-IU/ml (Roche Diagnostic Systems Inc, Mannheim, Germany). HBV genotyping was performed by direct sequencing of small-HBs-region (17). Anti-HBc was measured using the newly developed double-antigen sandwich immune-assay (Wantai, Beijing, China) calibrated using WHO standards (NIBSC, UK) as previously reported [17–19]. Total anti-HBc levels were reported in IU/mL. ALT were measured at each blood control and considered within the normal range when ≤1 x upper limit of normal (30 IU/L).

### Statistics

Data were expressed in median, range and percentiles values. Unpaired t-test and Whitney-U-test were used for total- and IgM-anti-HBc comparisons between groups. The Pearson-correlation was used for the correlation between total- and IgM-anti-HBc and other continuous variables. ROC analysis was used to analyze the efficacy of total- and IgM-anti-HBc to distinguish different HBsAg-carrier groups. Statistical analysis was performed using SPSS-17.0 software (SPSS, Chicago, IL, USA). All statistical analyses were based on 2-tailed hypothesis tests with a significance level of p<0.05.

## Results

**Fig 1:**
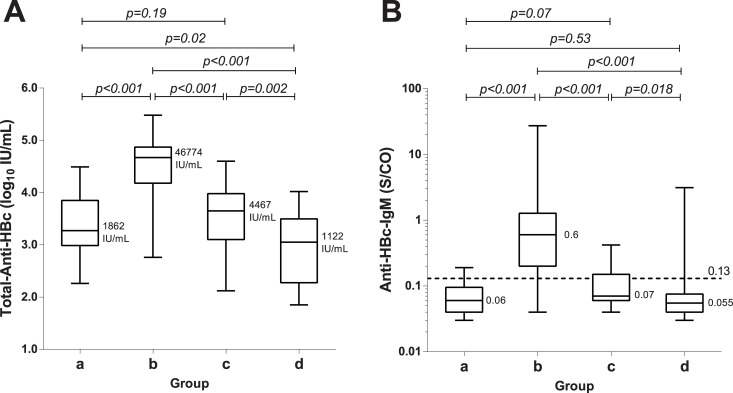
Box Plot analysis. Serum levels of total-anti-HBc (a) and anti-HBc-IgM (b) in 4 groups: a) inactive HBsAg-carriers; b) untreated HBeAg-negative-CHB patients, baseline; c) HBeAg-negative-CHB patients with SVR to Peg-IFN±NUC (EOF); d) NUC-treated patients (EOF). The distribution of total-anti-HBc levels falls within the dynamic range of quantification of the assay in all the patients. On the contrary, this holds true for 52% only of the sera, even using the lower CHB cut-off of 0.130 S/CO.

All sera tested positive for total-anti-HBc: their levels were significantly lower in HBsAg-carriers without HBV-induced liver disease (median 3.26, range 2.26–4.49Log_10_IU/ml) as compared to untreated CHB-patients (4.68,2.76–5.54Log_10_IU/ml) (p<0.0001), Fig 1. Total-anti-HBc levels were not significantly different in HBsAg-carriers without HBV-induced liver damage: immune-tolerant (1 case = 2.33Log_10_IU/ml), IC (3.09, 0.17–4.49Log_10_IU/ml) and CDH (2.70,0.17–3.99Log_10_IU/ml). In 6 IC who lost HBsAg during follow-up (146 months, range 120–216), total-anti-HBc levels showed a trend to be lower than in the remaining IC (2.75, 3.72–2.29Log_10_IU/ml versus 3.45, 4.11–2.26Log_10_IU/ml, p = 0.052). Total anti-HBc levels were not statistically different in untreated HBeAg-positive (4.71, 4.04–5.54Log_10_IU/ml) or HBeAg-negative (4.67,2.76–5.48Log_10_IU/ml) CHB patients.

Thirty of 212 (14.2%) sera from 27 of 111 (24.3%) HBsAg-carriers were IgM-anti-HBc positive using the acute-hepatitis-cut-off (1S/CO): 4 of 10 HBeAg-positive-CHB and 23 of 66 HBeAg-negative-CHB patients, but none of IC. According to the "chronic-hepatitis-cut-off" (0.130-S/CO) 102 of 212 sera (48.1%) were positive: all 9 HBeAg-positive-CHB patients, 51 of 66 (77.3%) HBeAg-negative-CHB, 5 of 25 (20%) IC and 1 of 10 (10%) CDH.

In HBsAg carriers without HBV-induced liver disease (IT, IC and CDH), IgM-anti-HBc (0.06, 0.03–0.61-S/CO) were significantly lower than in untreated CHB patients (0.53, 0.04–27.3-S/CO) (p<0.001). IgM-anti-HBc levels were comparable in HBsAg-carriers without HBV liver damage: 0.4-S/CO in the immune-tolerant carrier, mean values 0.07 0.03–0.19-S/CO in IC and 0.06, 0.03–0.19-S/CO in CDH. In 6 IC who lost HBsAg during follow-up IgM-anti-HBc levels were (0.03, 0.02–0.08-S/CO) not statistically different than in the remaining IC who remained HBsAg-positive (0.06, 0.03–0.19-S/CO). Similarly IgM-anti-HBc levels were not statistically different in untreated HBeAg-positive (0.61, 0.40–2.20-S/CO) and HBeAg-negative (0.60, 0.04–27.3-S/CO) CHB-patients.

**Fig 2:**
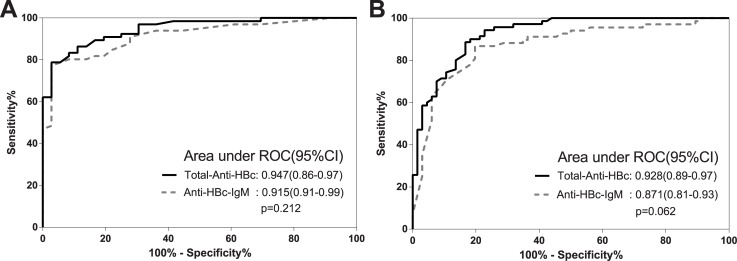
Receiver operating characteristic (ROC) curve analyses. Total-anti-HBc (full line) and anti-HBc-IgM (dotted line). A. AUROCs of total-anti-HBc and IgM-anti-HBc to identify IC from CHB were 0.947 (95% CI 0.86–0.97, p<0.0001) and 0.915 (95% CI 0.91–0.99, p<0.0001) respectively. B. AUROCs of total-anti-HBc and IgM-anti-HBc to identify HBeAg-negative-CHB with SVR from untreated HBeAg-negative-CHB were 0.928 (95% CI 0.89–0.97, p<0.0001) and 0.871 (95% CI 0.81–0.93, p<0.0001). The discriminative capacity of the 2 assays was comparable, even if total-anti-HBc performed better than IgM-anti-HBc (p = 0.212 and p = 0.062 for ROC analysis a and b respectively). We identified 2 cut-off-values for total-anti-HBc: the former (12489IU/ml) to distinguish IC from HBeAg-negative-CHB with 81.8%-sensitivity, 94.4%-specificity, 76.1%-PPV, 96.4%-NPVs and 87.3%-DA, the latter (10804IU/ml) to distinguish HBeAg-negative-CHB with SVR from untreated patients with 83.3%-sensitivity, 88.6%-specificity,84.9%-PPV, 87.3%-NPV and 86.0%-DA.

Overall total-anti-HBc and IgM-anti-HBc correlated significantly (p<0.001, r = 0.417) and both total-anti-HBc and IgM-anti-HBc correlated with ALT p<0.001, r = 0.351 and p = 0.008, r = 0.185 respectively. Using total-anti-HBc and IgM-anti-HBc as binary classifiers we ran two ROC curves separating: A) Inactive carriers (IC) from chronic-hepatitis-B (CHB) patients (Fig 2a: AUROC of 0.947 (95% CI 0.86–0.97, p<0.0001) for total-anti-HBc and 0.915 (95% CI 0.91–0.99, p<0.0001) for IgM-anti-HBc; B) Treated HBeAg-negative-CHB with SVR from untreated HBeAg-negative-CHB (Fig 2b: AUROCs of 0.947 (95% CI 0.86–0.97, p<0.0001) for total-anti-HBc and 0.915 (95% CI 0.91–0.99, p<0.0001) for IgM-anti-HBc.

### Kinetics of HBV markers patients treated with antivirals

In 57 HBeAg-negative-CHB patients treated with antivirals a progressive decline of total-anti-HBc levels was observed from BL (median values 4.67, range 2.76–5.48-Log_10_IU/ml) to EOF of SVR to Peg-IFN±NUCs or NUCs-treated patients (3.51, 1.85–5.16-Log_10_IU/ml, p<0.001).

In Peg-IFN±NUCs treated patients baseline total-anti-HBc levels were not significantly different in the 19 SVR (4.50, 2.76–5.17 Log_10_IU/ml), 10 REL (4.65, 3.30–5.15 Log_10_IU/ml) and 6 NR (4.23, 3.27–4.98). Similarly, at EOT total-anti-HBc were not significantly different in SVR(3.68, 2.65–4.27 Log_10_IU/ml), REL (3.88, 3.30–4.67 Log_10_IU/ml) and NR (3.95, 2.75–4.97-Log_10_IU/ml). At EOF in SVR total-anti-HBc levels were significantly lower (median 3.58, range 2.12–4.60 Log_10_IU/ml) as compared to baseline, p<0.001. In REL and NR, who were all shifted to NUC, total-anti-HBc levels at EOF vs BL were 4.21, 2.52–5.16 Log_10_IU/ml, p = 0.054 and 3.99, 2.66–5.16, p = 0.40 respectively.

At EOF in both Peg-IFN±NUCs and NUC-alone treated SVR-patients IgM-anti-HBc levels were lower than baseline (0.16, 0.03–3.13 vs 1.86, 0.04–27.3S/CO respectively, p<0.001; Fig 1).

**Fig 3:**
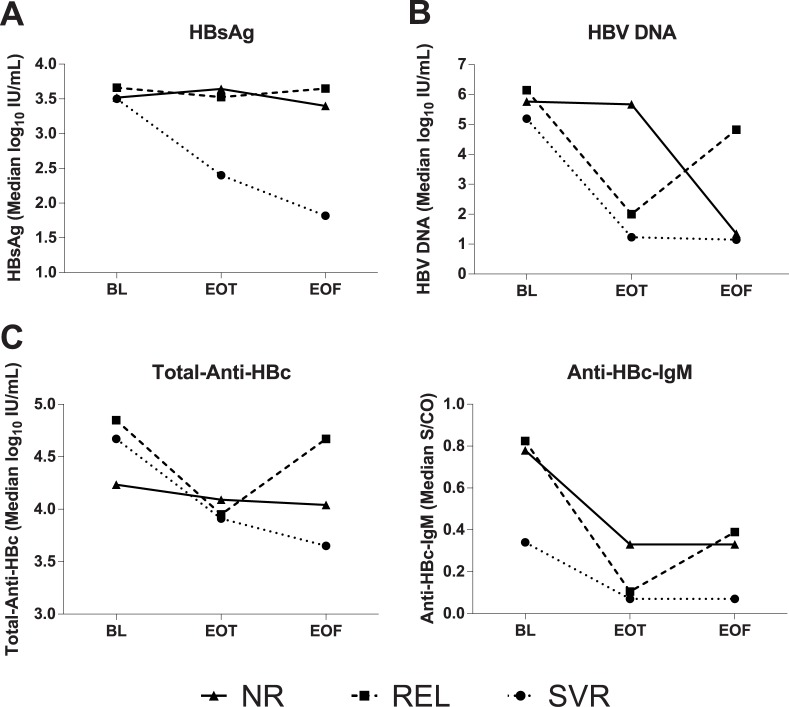
Kinetics of HBV markers in patients treated with Peg-IFN. Kinetics of median values of HBV markers between baseline (BL), end of therapy (EOT) and end of follow-up (EOF) in 35 patients treated with Peg-IFN: 6 NR, 10 REL and 19 SVR. Patients who did not respond to IFN (NR and REL) were subsequently switched to NUC. P values: a) HBsAg: BL vs EOT 0.27 for NR, 0.50 for REL and <0.001 for SVR; b) HBV-DNA: BL vs EOT 0.59 for NR, 0.014 for REL and <0.001 for SVR; c) Anti-HBc-IgM: BL vs EOT 0.24 for NR, 0.01 for REL and 0.17 for SVR; d) Total Anti-HBc: BL vs EOT 0.39 for NR, <0.001 for REL and <0.001 for SVR.

The variations of serum levels of HBsAg, HBV-DNA, total-anti-HBc, IgM anti-HBc from BL to EOT were compared in Peg-IFN±NUCs treated patients according to their treatment response (Fig 3). Total anti-HBc levels declined significantly in patients with sustained viral response, SVR (BL 4.50, 2.76–5.17-Log_10_IU/ml; EOT 3.68, 2.65–4.27-Log_10_IU/ml,p<0.001) or relapse, REL (BL 4.65, 3.79–5.15 Log_10_IU/ml; EOT3.80, 3.13–4.42-Log_10_IU/ml, p<0.001), but not in non-responders, NR (BL 4.23, 3.27–4.98 Log_10_IU/ml; EOT 4.00, 2.75–4.97-Log_10_IU/ml,p = 0.39).

IgM-anti-HBc declined significantly in REL (BL 1.16, 0.06–13.24, EOT 0.20, 0.03–1.22S/CO,p = 0.01) but not in SVR (BL 1.11, 0.04–3.6, EOT 0.13, 0.04–0.29S/CO,p = 0.172) and NR (BL 1.39, 0.19–4.99, EOT 0.41, 0.08–0.88S/CO,p = 0.238). HBV-DNA declined significantly in both SVR (BL 5.08, 0.00–8.04-Log_10_IU/ml, EOT1.54, 0.00–4.45-Log_10_IU/ml,p<0.001) and REL (BL 5.28, 2.0–7.62-Log_10_IU/ml, EOT 2.49, 0.78–5.13-Log_10_IU/ml,p = 0.014), but not in NR (BL 4.78, 0.00–8.13-Log_10_IU/ml, EOT 5.35, 0.00–9.73-Log_10_IU/ml,p = 0.59). Finally, HBsAg levels declined significantly in SVR only (BL 3.45, 1.13–4.7-Log_10_IU/ml, EOT 1.60, 0.7–3.9-Log_10_IU/ml,p = 0.001) but not in REL (BL 3.55, 2.58–4.07 Log_10_IU/ml, EOT 3.49, 2.37–4.14-Log_10_IU/ml,p = 0.496) and NR (BL 3.48, 2.88–3.96-Log_10_IU/ml, EOT 3.70, 3.29–4.23-Log_10_IU/ml,p = 0.267).

Overall, serum HBsAg and HBV-DNA the correlated with total anti-HBc significantly (p<0.001 r = 0.303 and p<0.001, r = 0.654 respectively), whereas they did not with IgM-anti-HBc (p = 075, r = 0.123 and p = 0.050, r = 0.135 respectively).

All HBeAg-negative-CHB patients (22) treated with NUC achieved both virologic (undetectable HBV-DNA) and biochemical (normal ALT) response. All serum markers of HBV infection declined significantly from BL to EOF: total-anti-HBc BL-median values of 4.58, 3.6–5.48-Log_10_IU/ml vs EOF 2.90, 1.85–4.02-Log_10_IU/ml,p<0.0001; IgM-anti-HBc BL 2.46, 0.04–27.3-S/CO vs EOF 0.20, 0.03–3.13S/CO,p<0.001; HBV-DNA, B 4.59, 0.7–8.11-Log_10_IU/ml vs EOF 0.27, 0.0–2.0-Log_10_IU/ml,p<0.0001; HBsAg BL3.27, 1.41–4.28 Log_10_IU/ml vs EOF 2.50, 0.03–3.13-Log_10_IU/ml,p = 0.0087.

**Fig 4:**
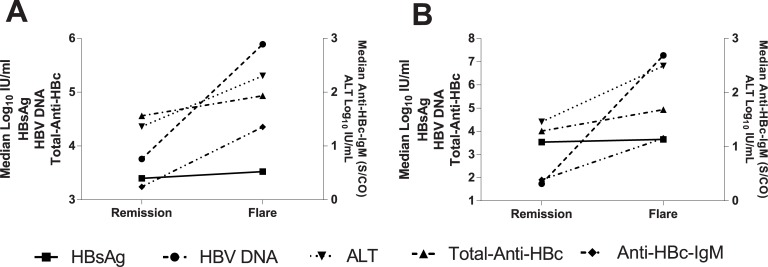
Kinetics of ALT and HBV markers between between remission and reactivation phases of CHB. A. untreated HBeAg-negative CHB patients at spontaneous disease remissions and flare-ups (5 cases): total-anti-HBc mean 4.23, median 4.56, and mean 4.88, median 4.93-Log_10_IU/ml, p = 0.054; IgM-anti-HBc mean 0.24, median 0.25, and mean 1.35, median 1.0S/CO, p = 0.054; HBV-DNA mean 3.78, median 3.76, and mean 5.86, median 5.89-Log_10_IU/ml, p = 0.058; HBsAg mean 3.18, median 3.4, and mean 3.21, median 3.52-Log_10_IU/ml, p = 0.91and ALT mean 23, median 23, and mean 244.6, median 201UI/L, p = 0.046. B. CHB patients treated with PEG-IFN (REL) at the time of temporary disease remission during therapy and hepatitis reactivation after relapse (9 cases): total-anti-HBc mean 4.04, median 4.01, and mean 4.79, median-4.93-Log_10_IU/ml, p = 0.002; IgM-anti-HBc mean 0.39, median 0.14, and mean 1.16, median 0.81S/CO, p = 0.03; HBV-DNA mean 3.34, median 1.74, and mean 7.18, median 7.27-Log_10_IU/ml, p = 0.004; HBsAg mean 3.61, median 3.54, and mean 3.55, median 3.65-Log_10_IU/ml, p = 0.73 and ALT mean 43, median 29, and mean 413, median 311 UI/L, p = 0.007.

Finally we studied the dynamic variations of ALT and HBV markers in 18 paired sera from 9 REL after Peg-IFN at the time of their on-treatment disease remission and hepatitis-B-relapse after treatment discontinuation and in 10 paired sera from 5 untreated HBeAg-negative-CHB patients with spontaneous remissions and reactivations. In Peg-IFN-REL all biomarkers showed statistically significant variations, but HBsAg, whereas in untreated patients only ALT variations were statistically significant whereas for total-anti-HBc, IgM anti-HBc and HBV-DNA there was a trend to significance (Fig 4).

## Discussion

The dynamic quantification range of the new total-anti-HBc assay allows to distinguish chronic-HBV-infection associated with HBV-induced liver disease, namely chronic-hepatitis-B (CHB), from chronic HBV infection without HBV-induced liver damage, namely non-inflammatory HBeAg-positive and inactive HBeAg-negative phases, independently from HBV-genotypes. Our validation study confirms in well characterized HBsAg-carriers infected with genotype-D- HBV the results obtained in Asian patients infected with genotype B and C [21]; the highest levels of total-anti-HBc were detected in CHB, both HBeAg-positive and HBeAg-negative and the lowest levels in inactive-carriers (Fig 1). Very low levels of the antibody were reported in the non-inflammatory, HBeAg-positive immune-tolerant-phase in genotype-B and-C infections [21–23]. Consistently in our HBeAg-positive-carriers total-anti-HBc levels comparable to those of inactive-carriers were found only in an immune-tolerant carrier, whereas the remaining CHB-patients showed high antibody levels. Total-anti-HBc declined during antiviral and correlated with response to Peg-IFN±NUC at EOT in both REL and SVR and at EOF in SVR during NUC treatment (Figs 1 and 3). In SVR-patients total-anti-HBc levels declined reaching at EOF values comparable to inactive-carriers. Interestingly in NUC-treated patients, the lower total-anti-HBc serum levels corresponded to the longer follow-up in disease remission. Antibody levels were significantly lower in NUC-treated patients) tested about 60 months after starting therapy as compared with patients with Peg-IFN SVR tested 24-weeks after EOT (p = 0.002). The distribution pattern of IgM-anti-HBc in the same subgroups mimics that of total-anti-HBc, but unfortunately the dynamic quantification range of the IgM-assay hampers the diagnostic accuracy since half of values fall below the analytical specificity cut-off of the assay. Comparing the kinetics of the different HBV-markers according to response to Peg-IFN we found (Fig 3) that the decline of total-anti-HBc during therapy parallels that of HBV-DNA and this may be useful in clinical practice to confirm the effectiveness of Peg-IFN antiviral activity. However, during Peg-IFN-treatment total-anti-HBc is unable to distinguish REL from SVR who are instead identified better by HBsAg-kinetics as reported previously [17–20]. During NUC therapy the kinetics of total anti-HBc differ from those of the other HBV markers and the total-anti-HBc decline provides an added value to HBV-DNA and HBsAg monitoring beckoning the remission of HBV-induced liver damage under effective antiviral therapy. In patients treated with Entecavir or Tenofovir viral load is consistently suppressed in the very early phase of treatment, whereas HBsAg declines very slowly over time [17–20]. Interestingly long-term NUC-treated patients with very low levels of total-anti-HBc experienced the HBsAg-clearance with anti-HBs-sero-conversion during follow-up. Thus, quantification of total-anti-HBc might help to predict HBsAg loss and future long-term prospective studies should address the issue in larger cohort of patients. Finally, the findings that total-anti-HBc kinetics correlate with ALT levels as well as IgM-anti-HBc in both naturally-occurring [25–27] and therapy-induced remission and reactivation phases [26,27] support the view that total-anti-HBc is a reliable marker of HBV-induced liver disease in chronic HBsAg-carriers. In chronic HBeAg positive hepatitis B baseline total-anti-HBc levels were shown to predict HBeAg to anti-HBe seroconversion in Asiatic HBeAg-positive-CHB patients, treated with Peg-IFN or NUC infected with HBV genotype B and C [24]. Our findings underline the potential of the assay also in the management of chronic HBeAg-negative-CHB of the Mediterranean Area infected with HBV genotype D. In addition our work suggests that quantification of total-anti-HBc may be helpful to distinguish the inactive HBsAg carrier from HBeAg-negative-CHB which is characterized by intervening phases of disease remission and reactivation [25–26], to identify patients with higher chance of HBsAg clearance and to provide a new tool to answer the unmet needs for treatment tailoring [27–30].

All available data support the view that q-anti-HBc is complementary to HBsAg quantification. HBsAg is a product of HBV replication, ccc-HBV-DNA transcription and viral mRNAs translation whereas total-anti-HBc is expression of the antiviral immune response against the HBV “core” antigen. Our data suggest that symmetry or asymmetry of the two markers may have important diagnostic implications: high levels of HBsAg associated with low levels of total-anti-HBc are diagnostic for the immune-tolerance or florid non-inflammatory phase of HBeAg-positive HBV-infection, while high levels of both markers identify chronic hepatitis B (either HBeAg-positive and HBeAg-negative). In cirrhotic patients with advanced HBeAg-negative-CHB total-anti-HBc levels are high because of persistent HBV-induced liver disease whereas HBsAg may decline because of HBsAg deletion mutants which hamper HBsAg secretion. In treated patients the decline of total-anti-HBc parallel HBsAg-kinetics in patients who respond to anti-viral treatment (sustained responders to therapy), but is asymmetric with the unchanged HBsAg levels in patients whose hepatitis B recurs after treatment discontinuation (Relaspers). In addition the decline of HBsAg during antiviral therapy is HBV genotype dependent whereas the decline of total-anti-HBc is HBV genotype independent. Thus the combined used of both markers can improve the management of the HBsAg carrier consistently.

In conclusion total-anti-HBc as measured by double-antigen-sandwich-immune-assay is a reliable non invasive marker of HBV-induced liver disease helpful to identify chronic-HBV-infection associated with HBV-induced liver disease. Larger prospective studies are needed address to address its significance particularly in the clinical management of NUC-treated patients.

## Supporting Information

S1 Data The results of all HBV markers in the 212 sera panel are reported in an Excel format. (XLS)
